# State Medical Society

**Published:** 1881-02

**Authors:** L. F. Worrell

**Affiliations:** Chairman of the Committee on Necrology; Bloomington, Ill.


### ARTICLE X.

BLOOMINGTON, ILL., Jan. 12, 1881.

The members of the Illinois State Medical Society are hereby requested to report to me or to Dr. G. W. Alvin, or to Dr. E. Ingals, the name of any member of our society who has died during the last year, together with such biography as they may be able to furnish.

L. F. WORRELL,
*Chairman of the Committee on Necrology.*

